# Smart Tetraphenylethene‐Based Luminescent Metal–Organic Frameworks with Amide‐Assisted Thermofluorochromics and Piezofluorochromics

**DOI:** 10.1002/advs.202200850

**Published:** 2022-03-31

**Authors:** Zhong‐Hong Zhu, Changjiang Bi, Hua‐Hong Zou, Guangxue Feng, Shuping Xu, Ben Zhong Tang

**Affiliations:** ^1^ State Key Laboratory of Luminescent Materials and Devices Guangdong Provincial Key Laboratory of Luminescence from Molecular Aggregates School of Materials Science and Engineering South China University of Technology Guangzhou 510640 China; ^2^ State Key Laboratory of Supramolecular Structure and Materials Institute of Theoretical Chemistry College of Chemistry Jilin University Changchun 130012 China; ^3^ State Key Laboratory for Chemistry and Molecular Engineering of Medicinal Resources School of Chemistry and Pharmacy of Guangxi Normal University Guilin 541004 China; ^4^ School of Science and Engineering Shenzhen Institute of Aggregate Science and Technology The Chinese University of Hong Kong Shenzhen 518172 China

**Keywords:** aggregation‐induced emission, host–guest interactions, luminescent metal–organic frameworks, piezofluorochromics, thermofluorochromics

## Abstract

Luminescent metal–organic frameworks (MOFs) are appealing for the design of smart responsive materials, whereas aggregation‐induced emission (AIE) fluorophores with twisted molecular rotor structure provide exciting opportunities to construct MOFs with new topology and responsiveness. Herein, it is reported that elongating AIE rotor ligands can render the newly formed AIE MOF (ZnETTB) (ETTB = 4',4''',4''''',4'''''''‐(ethene‐1,1,2,2‐tetrayl)tetrakis(([1,1'‐biphenyl]‐3,5‐dicarboxylic acid))) with more elasticity, more control for intramolecular motion, and specific amide‐sensing capability. ZnETTB shows specific host–guest interaction with amide, where *N,N*‐diethylformamide (DEF), as an example, is anchored through C—H···O and C—H···*π* bonds with Zn cluster and ETTB^8−^ ligand, respectively. DEF anchoring reduces both the distortion level and the intramolecular motions of ETTB^8−^ ligand to lead a blueshifted and intensified emission for DEF ∈ ZnETTB. Moreover, amide anchoring also affords the DEF ∈ ZnETTB with the excellent thermofluorochromic behavior, and further increases the piezofluorochromic sensitivity at low‐pressure ranges on the basis of its elastic framework. This work is one of the rare examples of amide‐responsive smart materials, which shall shed new lights on design of smart MOFs with twisted AIE rotors for further sensing and detection applications.

## Graphical abstract

It is reported that elongating aggregation‐induced emission (AIE) rotor ligand can render the newly formed AIE metal–organic framework (MOF) (ZnETTB) with more elasticity, more control for intramolecular motion, and specific amide‐sensing capability. Amide anchoring further yields the resultant MOF with thermofluorochromic behavior and improved piezofluorochromic sensitivity.

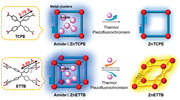

## 1 Introduction

Metal–organic frameworks (MOFs) are well‐defined crystalline porous materials with periodic network structure formed by alternately connecting the second building units (SBUs) and organic ligands.^[^ ^1^ ^]^ A variety of MOFs have been developed to show promising performance in many fields, such as gas storage and separation, catalysis, solid‐state lighting, drug delivery, and sensing.^[^ ^2^, ^3^, ^4^, ^5^ ^]^ Among them, smart luminescent MOFs have emerged as a new class of optical materials, which marry the merits of void space of porous framework and ultrasensitivity of fluorescence technique.^[^ ^6^, ^7^, ^8^ ^]^ The luminescence could originate from the organic ligands, SBUs, or guest fluorophores in the pores, whereas all could be designed to show luminescence changes when external stimuli trigger structural transformation, molecular alignment, or framework collapse, etc.^[^ ^9^, ^10^ ^]^ More importantly, the controllable topology and pore sizes further facilitate specific molecular recognition and host–guest interaction for improved responsiveness.^[^ ^11^ ^]^ As a consequence, smart luminescent MOFs have delivered excellent performance in solvo‐/piezo‐fluorochromism, gas detection, data storage, drug delivery monitoring, and so on, presenting a promising class of novel smart materials.^[^ ^11^, ^12^, ^13^, ^14^, ^15^ ^]^

Transplanting dynamic motifs such as organic ligands with moving moieties (e.g., rotary, vibrational, or elastic elements) into chemically rigid frameworks to derive artificial molecular machinery has been a transformative approach for designing novel smart materials.^[^ ^16^, ^17^, ^18^, ^19^, ^20^, ^21^, ^22^, ^23^, ^24^, ^25^ ^]^ As compared to conventional luminescent MOFs, the presence of these molecular machinery elements provides attentional and unprecedented responsiveness. Aggregation‐induced emission (AIE) fluorogens (AIEgens) are a novel class of organic fluorophores, whose emission is largely associated with their twisted molecular geometry and degree of intramolecular motions.^[^ ^26^, ^27^, ^28^ ^]^ AIEgens usually exhibit weak or even diminished emission in molecular state but show largely boosted emission in aggregate state when intramolecular motions are largely inhibited.^[^ ^29^, ^30^ ^]^ The twisted molecular geometry provides great opportunities for designing smart luminescent MOFs with new topology and elasticity.^[^ ^31^ ^]^ Moreover, the porous framework provides free volume to modulate the degrees of structural distortion, intramolecular motion, and interaction by external stimuli to deliver smart responsiveness.^[^ ^32^, ^33^, ^34^ ^]^ For example, Zhou and co‐workers successfully constructed an elastic AIE‐MOF (PCN‐128W) with piezofluorochromic behavior.^[^ ^20^ ^]^ Zhao and co‐workers introduced a flexible tetraphenylethylene (TPE) linker to the mesoporous MOF (NUS‐13) with minimal rotation resistance for sensing.^[^ ^18^ ^]^ Shi and co‐workers reported a TPE‐based MOF to show reversible fluorescence switch in response to temperature and pressure due to the deformation of crystal structures.^[^ ^35^ ^]^ Bu and co‐workers also reported a thermofluorochromic MOF (NKU‐128) by using tri(4‐(pyridine‐3‐yl)phenyl)amine (3‐TPPA), capable of conformational change as the ligand.^[^ ^36^ ^]^ Recently, we also demonstrated that MOF with differently charged SBUs could be obtained with twisted AIE ligand, which exhibited specific HCl‐vapor‐induced fluorescence and magnetism switch.^[^ ^31^ ^]^ Therefore, AIEgens with twisted molecular geometry and molecular rotors shall be superb candidates to design novel luminescent MOFs with smart responsiveness.^[^ ^22^, ^23^, ^32^, ^33^, ^34^, ^35^, ^36^ ^]^

**Scheme 1:**
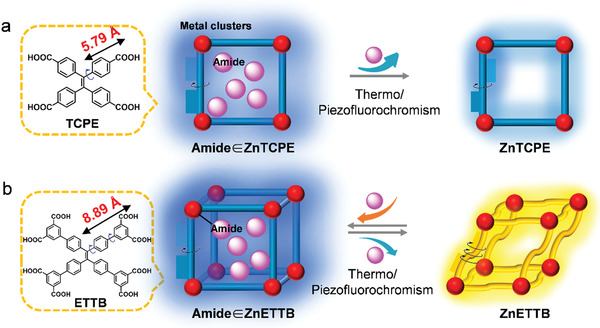
Schematic illustration thermo‐/piezofluorochromic AIE MOFs (Solv. ∈ ZnTCPE and Solv. ∈ ZnETTB (Solv. = amide)) constructed by a) H_4_TCPE and b) H_8_ETTB, respectively.

Herein, we report an amide‐responsive luminescent AIE MOF (ZnETTB) built with elongated AIE ligand, and demonstrate that the incorporation of amide in framework could afford the MOF with excellent thermofluorochromic behavior and largely improved piezofluorochromic sensitivity. In specific, elongation of TPE‐derived ligand from 4,4′,4″,4‴‐(ethene‐1,1,2,2‐tetrayl)tetrabenzoic acid (H_4_TCPE) to 4′,4‴,4′′′′′,4′′′′′′′‐(ethene‐1,1,2,2‐tetrayl)tetrakis([1,1′‐biphenyl]‐3,5‐dicarboxylic acid) (H_8_ETTB) could readily convert the 2D ZnTCPE to 3D ZnETTB. Such ligand elongation increases frame elasticity and provides void space for intramolecular motion, and the resultant ZnETTB showed reduced rather than enhanced fluorescence as compared to H_8_ETTB powders. The ligand elongation also introduces amide responsiveness to ZnETTB (**Scheme** **1**), where the amide guest (e.g., *N,N*‐diethylformamide (DEF)) is anchored by strong C—H···O hydrogen bonds with SBUs and C—H···*π* bonds with ETTB^8−^ ligands. Such amide anchoring reduces distortion level of ETTB^8−^ as well as the degree of intramolecular rotation, resulting in hypochromatic shift of emission wavelength (over 108 nm) with largely intensified brightness. The gradually loss of amide at ascending temperature renders the Solv. ∈ ZnETTB with the excellent thermofluorochromic behavior, which shows linearly redshifted emission (437–530 nm) and descended brightness in the temperature range of 20–160 °C, significantly outperforming Solv. ∈ ZnTCPE (450–470 nm). With longer ligand length and elastic frame, DEF ∈ ZnETTB also shows better piezofluorochromic behavior over DEF ∈ ZnTCPE. More importantly, amide anchoring significantly increases piezofluorochromic sensitivity of DEF ∈ ZnETTB at low‐pressure ranges. This work demonstrates the unique merits of AIEgens with twisted geometry and molecular machinery elements in the design of novel MOFs with new topology, elasticity, and unprecedented responsiveness, which shall shed new light for the further development of smart MOF‐based sensors.

## 2 Results and Discussion

### 2.1 Synthesis and Characterization of AIE MOFs

**Figure 1:**
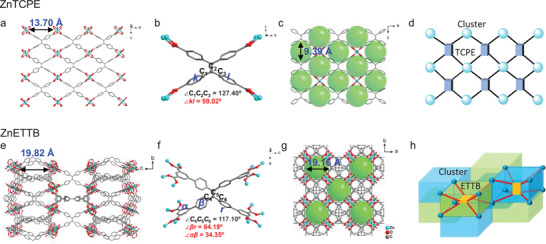
Structures of a,c) ZnTCPE and e,g) ZnETTB. Molecular geometries of b) TCPE^4−^ and f) ETTB^8−^ in the ZnTCPE and ZnETTB structures. Topological connection of d) ZnTCPE and h) ZnETTB.

Two rotary TPE derivatives, H_4_TCPE and its elongated derivative H_8_ETTB, were synthesized and selected as the twisted AIE ligands to coordinate with Zn(II) clusters to form AIE MOFs. ZnTCPE was first synthesized through the solvothermal reaction between H_4_TCPE and Zn(NO_3_)_2_·6H_2_O.^[^ ^37^ ^]^ Single crystal X‐ray diffraction (SCXRD) analysis reveals that ZnTCPE crystallizes in the *P*2_1_/*c* space group of the monoclinic system (Tables S1 and S2, Supporting Information). ZnTCPE showed staggered 2D MOF sheets made from paddlewheel‐shaped Zn_2_(TCPE)_4_ SBUs bridged by TCPE^4−^ ligands (**Figure** **1**a–d). The dihedral angle of the twisted TCPE^4−^ in the ZnTCPE structure is ∠*kl* = 59.02°, and the angle of *kl* connecting carbon atoms is ∠C_1_C_2_C_3_ = 127.40° (Figure 1b). The carboxylic acid coordination sites in H_4_TCPE are all located in the para position of the four benzene rings and in the same plane; so, the obtained ZnTCPE has 2D topological connections (Figure 1d). Polycrystalline powder X‐ray diffraction (PXRD) shows that the Solv. ∈ ZnTCPE (Solv. are free solvent molecules, which are determined to be DEF later) is a pure phase (Figure S1, Supporting Information). The thermogravimetric (TG) analysis in N_2_ atmosphere revealed a 28.60% weight loss for Solv. ∈ ZnTCPE before 210 °C, corresponding to the loss of three DEF molecules in the pores (Figure S2, Supporting Information). Solv. ∈ ZnTCPE showed the same solid‐state fluorescence spectrum as its H_4_TCPE ligand but with largely enhanced brightness due to the coordination‐restricted intramolecular motion of H_4_TCPE (Figure S3, Supporting Information). The activated ZnTCPE showed a slightly redshifted emission maximum (≈20 nm) with reduced fluorescence intensity as loss of DEF increases the degree of intramolecular motion of H_4_TCPE, which is still much higher than its H_4_TCPE ligand, suggesting the rigid framework of ZnTCPE.

Elongating H_4_TCPE with one additional phenyl ring on each periphery, the more flexible and softer H_8_ETTB was synthesized to construct the porous ZnETTB with a more complicated topology. The PXRD value of Solv. ∈ ZnETTB is consistent with the simulated value, indicating its pure phase (Figure S4, Supporting Information). The infrared (IR) characteristic absorption peaks of Solv. ∈ ZnTCPE are located at 3415 cm^−1^ (s, —OH, and H_2_O), 1645 cm^−1^ (s, —COO^−^, and —C═O), and 1385 cm^−1^ (s and —C═C—), respectively (Figure S5, Supporting Information). The scanning electron microscopy (SEM) images showed that the obtained Solv. ∈ ZnETTB is a block crystal (Figure S6, Supporting Information). TG analysis in N_2_ atmosphere revealed a 40.77% weight loss for Solv. ∈ ZnETTB before 300 °C, corresponding to four DEF molecules in the pores (Figure S7, Supporting Information). The activated ZnETTB was further obtained by heating to remove DEF. The saturated N_2_ uptake of ZnETTB (503 cm^3^ g^−1^) corresponds to the Brunauer–Emmett–Teller (BET) surface area of 817.1785 m^2^ g^−1^ (Figure S8, Supporting Information), indicating the porous nature of ZnETTB. SCXRD result further indicates that ZnETTB crystallizes in the *P*‐42*c* space group of the tetragonal crystal system (Figure 1e; Tables S3 and S4, Supporting Information), and it only contains one type Zn cluster SBU with a pyramidal coordination configuration. Each carboxylate group of one ETTB^8−^ ligand is linked to one Zn_2_‐paddlewheel SBU to form an independent unit, and each ETTB^8−^ is coordinated with 16 Zn(II) ions (8 Zn_2_ clusters) (Figure 1e–h). Two Zn_2_ clusters and ETTB^8−^ with different orientations are alternately connected to form a 3D porous structure. These eight carboxylic acid coordination sites in H_8_ETTB are located in the meta position of the *α*‐benzene ring, and have opposite orientations out of the main plane, which leads to the effective formation of 3D topology for ZnETTB (Figure 1h). ZnETTB possesses a larger pore size of 19.16 Å and a more complicated 3D topological structure as compared to ZnTCPE (Figure 1g). The ETTB^8−^ in the ZnETTB has a larger dihedral angle of ∠*βr* = 84.19° than TCPE^4−^ in ZnTCPE, and the angle of connecting carbon atoms is ∠C_4_C_5_C_6_ = 117.10° (Figure 1f). In addition, the ETTB^8−^ in the ZnETTB structure also has a dihedral angle of ∠*αβ* = 34.35° between the *α* (the additional phenyl ring) and *β* planes. It should be noted that although SCXRD reveals the optimized angels for ∠*αβ* and ∠*βr*, there is minimal resistance to block the rotation of *β* ring.

The optical properties of ZnETTB and Solv. ∈ ZnETTB were further investigated. Intriguingly, ZnETTB showed redshifted and reduced emission as compared to H_8_ETTB powder, and its solid‐state fluorescence wavelength is redshifted by 78 nm (Figure S9, Supporting Information). Such an unprecedented phenomenon was quite different from these reported AIE MOFs where largely enhanced emission was often observed.^[^ ^35^, ^37^ ^]^ The reduced emission intensity of ZnETTB shall be mainly caused by the elongation of AIE ligands, which provides more space for intramolecular motions of ETTB^8−^ to nonradiatively dissipate excited energy. On the contrary, Solv. ∈ ZnETTB showed blueshifted and largely enhanced emission as compared to both ZnETTB and H_8_ETTB powders. The emission peaks for Solv. ∈ ZnETTB, H_8_ETTB, and ZnETTB are located at 437, 470, and 548 nm, respectively. The higher and blueshifted emission for Solv. ∈ ZnETTB shall be attributed to presence of guest solvent molecules in the MOF pores, which immobilizes the ETTB^8−^ ligands and prevents the excited state distortion of the ETTB^8−^, resulting in blueshifted and enhanced emission. It should also be noted that ZnETTB showed excellent stability as evidenced by nearly unchanged emission spectra after being placed in the air for a month.

### 2.2 Thermofluorochromic Behaviors of AIE MOFs

**Figure 2:**
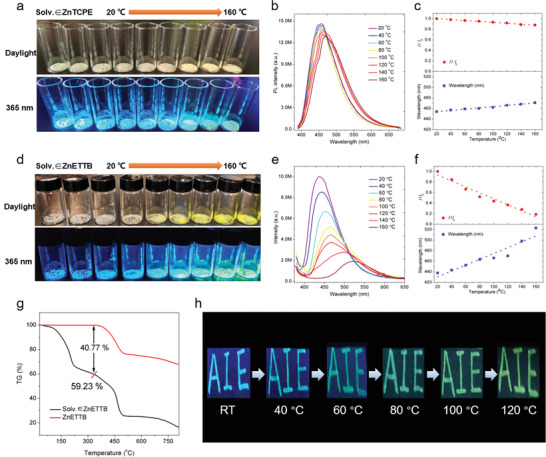
Photos of a) Solv. ∈ ZnTCPE and d) Solv. ∈ ZnETTB under daylight and 365 nm UV lamps after being treated under different temperatures for 2 h. The solid‐state fluorescence spectra of b) Solv. ∈ ZnTCPE and e) Solv. ∈ ZnETTB upon heating under different temperatures for 2 h. Linear fitting of emission intensity (*I*/*I*_0_) and wavelength of c) Solv. ∈ ZnTCPE and f) Solv. ∈ ZnETTB versus temperature. g) The TG curves of Solv. ∈ ZnETTB and ZnETTB. h) The photographs of handwrote “AIE” with Solv. ∈ ZnETTB under UV irradiation upon increasing temperatures.

Considering the large emission difference between ZnETTB and Solv. ∈ ZnETTB, we hypothesize that the release of guest DEF solvent molecules in response to high temperature could be used for thermofluorochromics. Herein, we tested the fluorescence changes of Solv. ∈ ZnTCPE and Solv. ∈ ZnETTB in the temperature range of 20–160 °C under atmospheric condition. The Solv. ∈ ZnTCPE crystals show negligible color change and emission change under room light and 365 nm UV lamps, respectively (**Figure** **2**a). Further solid‐state fluorescence spectrum test showed that the emission wavelength of Solv. ∈ ZnTCPE slightly redshifted from 450 to 470 nm, accompanied with a 13.4% reduction in fluorescence intensity when increasing temperature (Figure 2b). Although Solv. ∈ ZnTCPE only shows weak thermochromic behavior, it still gave a good linear correlation between its solid‐state fluorescence intensity (*I*/*I*_0_) or wavelength change and temperature change (Figure 2c). In addition, Solv. ∈ ZnTCPE and ZnTCPE showed similar fluorescence lifetimes of 3.94 and 4.14 ns, respectively (Figure S10, Supporting Information), suggesting that a minimal difference was introduced by solvent molecules for ZnTCPE. On the contrary, Solv. ∈ ZnETTB gradually switched from white to yellow powder with a blue‐to‐green emission transition along with increased temperature (Figure 2d). The solid‐state emission peak of Solv. ∈ ZnETTB redshifted from 437 to 530 nm, and the emission peak intensity is reduced by ≈5.6‐fold (1.0 × 10^7^–1.8 × 10^6^ a.u.) (Figure 2e). The chromaticity coordinate (CIE) also shows a large degree of solid‐state fluorescence color change for Solv. ∈ ZnETTB in the temperature range of 20–160 °C (Figure S11, Supporting Information). Moreover, Solv. ∈ ZnETTB also showed a very good linear relationship between emission peak wavelength (or intensity) and temperature. The thermofluorochromic sensitivity of Solv. ∈ ZnETTB was determined to be 0.67 nm °C^−1^, which is much higher than Solv. ∈ ZnTCPE (0.14 nm °C^−1^), suggesting that Solv. ∈ ZnETTB serves well the purpose of thermochromic sensor in the range of 20–160 °C (Figure 2f). In addition, the unchanged UV–vis and IR absorption spectra before and after the thermochromic test were observed, indicating the excellent stability of Solv. ∈ ZnETTB, which also hints that such thermofluorochromic is induced solely by the free guest DEF molecule (Figures S12 and S13, Supporting Information). Solv. ∈ ZnETTB exhibited nearly unchanged PXRD results after different temperature treatment, further indicating its excellent frame structure stability during the above heating process (Figure S14, Supporting Information). The diffraction peak around 10° gradually disappears when temperature increases, which shall be due to the increased free space around the ETTB^8−^ ligand as a result of solvent loss, resulting in a relative more disordered structure (Figure S14, Supporting Information). In addition, Solv. ∈ ZnETTB demonstrated excellent thermofluorochromic reliability, whose emission repeatedly switched between Solv. ∈ ZnETTB and ZnETTB for many cycles (Figure S15, Supporting Information). TG analysis also reveals that the presence of absorbed solvent molecules (namely DEF) is the main difference between Solv. ∈ ZnETTB and ZnETTB (Figure 2g). These data collectively implied that free solvent molecules in the pore are the main reason for themofluorochromism. As mentioned earlier, ETTB^8−^ is a typical AIE fluorophore; the vibration and rotation of ETTB^8−^are restricted to a certain extent when the Solv. ∈ ZnETTB pores are filled with free solvent molecules, leading to enhanced fluorescence for Solv. ∈ ZnETTB as compared to ZnETTB. At a high temperature, the loss of DEF in the pores will increase the softness of the main frame structure and provide more space for intramolecular rotation of ETTB^8−^. As a consequence, the free vibration and rotation of ETTB^8−^ lead to the redshifted but weakened fluorescence. On the other hand, Solv. ∈ ZnTCPE possesses strong rigidity as the phenyl rings of TCPE^4−^ are restricted by the Zn_2_ clusters; it becomes difficult to alert the framework and intramolecular motions to a large amplitude by the entry and loss of guest molecules. Therefore, Solv. ∈ ZnTCPE demonstrates poor thermofluorchromic behavior. Such excellent thermochromic behaviors also yield Solv. ∈ ZnETTB with the temperature‐sensitive anticounterfeiting ability. After Solv. ∈ ZnETTB is ultrasonically dispersed, the solution is used as anticounterfeiting ink. The handwritten “AIE” was gradually shifted from blue emission to yellow emission upon heating, which renders the ink the capability to hide information and release such secured information at certain conditions for anticounterfeiting applications (Figure 2h).

### 2.3 Specific Luminescence Responsiveness toward Amide

**Figure 3:**
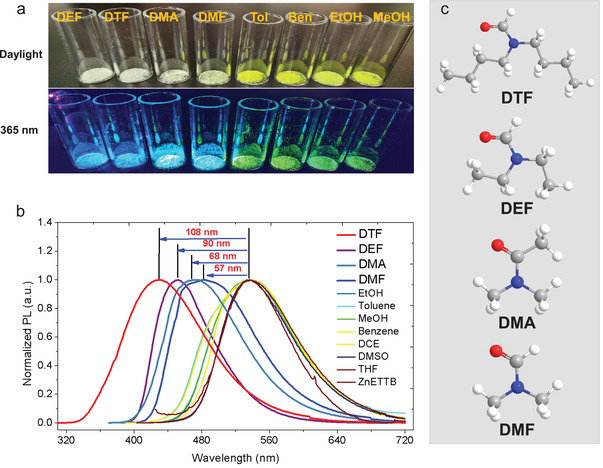
a) Photos of ZnETTB crystals under daylight and 365 nm UV lamps after being treated under different organic solvents. b) Solid‐state fluorescence spectra of ZnETTB after soaking in different organic solvents. c) Chemical structures of selected amide molecules.

The molecular geometry and intramolecular motions of AIE ligands in MOFs could be easily disturbed by the guest molecules in the pores, eventually leading to fluorescence changes. The thermochromic behavior of Solv. ∈ ZnETTB was attributed to the presence of DEF solvent molecules in the pores, which further motivated us to investigate the specificity of such fluorescence responsiveness toward other molecules. The activated ZnETTB was socked in different commonly used solvents (e.g., *N,N*‐dimethylformamide (DMF), dimethyl sulfoxide (DMSO), tetrahydrofuran (THF), and EtOH) for 30 min, and the solid‐state fluorescence behavior was monitored after these MOF crystals are collected by centrifugation and filtration. It is worth noting that the amide solvent socking leads to a significant fluorescence change for ZnETTB, which switched from yellow crystals to white crystals under daylight, with a green‐to‐blue emission switch under UV light, while all other solvents could not cause any observable changes to the appearance and emission of ZnETTB crystals (**Figure** **3**a). Such fluorescence changes were further confirmed by solid‐state fluorescence spectrum measurement. Only amide molecules could change the emission spectra of ZnETTB, and its emission peak was blueshifted by 57, 68, 90, and 108 nm when the socking solvents are DEF, DMF, *N,N*‐dimethylacetamide (DMA), and *N,N*‐dibutylformamide (DTF), respectively (Figure 3b,c). In addition, PXRD spectra of ZnETTB show minimal changes after soaking in different organic solvents, indicating its high stability (Figure S16, Supporting Information). Amide solvents, as important industrial raw materials, are raw materials, intermediates, reaction media, and crystallization solvents for pharmaceutical synthesis and dye production. Toxicological studies have confirmed that amide solvents can be absorbed into the body through the respiratory tract, digestive tract, and skin and mucous membranes, and their metabolic rate in the organism is low. Therefore, the development of intelligently responsive compounds to high‐boiling amide solvent molecules is very important, but progress is still slow so far. Our discovery shall pave new steps for development amide sensors.

**Figure 4:**
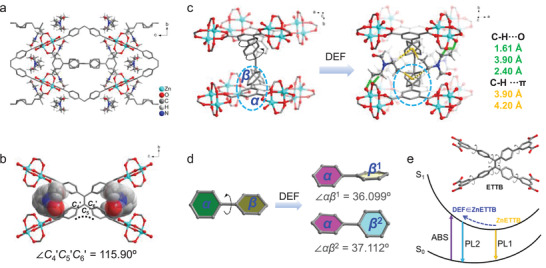
a) The SCXRD structure of DEF ∈ ZnETTB. b) The distribution of anchored DEF molecules around the ligand ETTB^8−^. c) The structural difference between ZnETTB and DEF ∈ ZnETTB. d) The change of the angle between the *α* and *β* phenyl rings on the ETTB^8−^ ligands in the structure of ZnETTB and DEF ∈ ZnETTB. e) Proposed fluorescence decay paths in ZnETTB (path PL1) and DEF ∈ ZnETTB (path PL2).

To further explore the mechanism of the specific amide responsiveness of ZnETTB, we soaked ZnETTB in DEF and obtained the single crystal information of DEF ∈ ZnETTB. ZnETTB and DEF ∈ ZnETTB exhibited very similar crystal unit cell parameters, where DEF ∈ ZnETTB yielded slightly increased lengths in *a‐* and *b*‐directions, due to the incorporation of DEF in the pore (Table S5, Supporting Information). However, they belong to different crystalline groups, ZnETTB crystallize in *P*‐42*c* space group, while DEF ∈ ZnETTB crystallizes in the *P*4_2_/*mnm* space group of the tetragonal crystal system (Tables S5 and S6, Supporting Information). Such a transformation indicates that DEF introduces new symmetries to the MOF. In details, ZnETTB belongs to the *D*_2_ *_d_* point group with *S*_4_, 2*C*_2_, and 2*σ* symmetric operations, while DEF ∈ ZnETTB belongs to the *D*_4_ *_h_* point group with *C*_4_, 4*C*_2_, and 4*σ* symmetric operations. In the DEF ∈ ZnETTB structure, at least four DEF molecules are distributed around the periphery of each ETTB^8−^ ligands (**Figure** **4**a,b). The presence of DEF molecules disturbed the geometry of ETTB^8−^ ligand. The distortion angel of ∠C_4_C_5_C_6_ in ZnETTB (117.10°) was reduced to 115.90° for new ∠C_4_′C_5_′C_6_′ in DEF ∈ ZnETTB, indicating a less distorted geometry of ETTB^8−^ ligand with the presence of DEF guests (Figure 4b). Further SCXRD analysis indicated that the amide guest DEF is anchored by strong hydrogen bond, C—H···O (1.61, 3.90, and 2.40 Å), with Zn clusters, as well as the additional C—H···*π* (3.90 and 4.20 Å) bond between DEF and the *β* phenyl ring of ETTB^8−^ (Figure 4c). The *β* benzene rings in the porous ZnETTB framework with minimal rotation resistance can rotate freely under the excited state condition, leading to an increase in nonradiative dissipation (Figure 4c,d); hence, ZnETTB showed reduced emission as compared to its H_8_ETTB ligand. The anchored DEF molecules introduce the C—H···*π* bonds to the *β* benzene rings, which increased the rotation resistance of the *β* benzene ring. Two possible dihedral angles (36.099° and 37.112°) between the *α* and *β* benzene rings in DEF ∈ ZnETTB were identified, indicating the largely reduced intramolecular rotation for ETTB^8−^ ligand in MOF (Figure 4d). Therefore, the specific DEF anchoring is responsible for the blueshifted and largely enhanced emission of DEF ∈ ZnETTB over ZnETTB. The insertion of DEF introduces the steric hindrance to ETTB^8−^, which prevents the excited state distortion of ETTB^8−^, resulting in a high‐energy excited state conformation. As a result, DEF ∈ ZnETTB possesses a larger excited–ground state energy gap, hence a shorted emission wavelength as compared to ZnETTB. In addition, the guest DEF inhibits the rotation of *β* benzene rings through the C—H···*π* effect, which effectively enhances the restriction of intramolecular motions (RIM) effect in the excited state, which leads to largely enhanced emission for DEF ∈ ZnETTB. The increased lifetime for DEF ∈ ZnETTB (1.92 ns, PL2) over ZnETTB (3.79 ns, PL1) further confirmed that DEF supports the framework structure of the MOF, placing it in a high‐energy excited state (Figure S17, Supporting Information). Therefore, DEF ∈ ZnETTB could dissipate exciton energy through a faster radiative decay process than ZnETTB, resulting in a blueshifted emission and shorted fluorescence lifetime (Figure 4e).^[^ ^38^ ^]^ The release of DEF molecules under high temperature affords the phenyl rings of ETTB^8−^ a certain degree of freedom to rotate as well as a relatively distorted flexible porous framework, leading to redshifted but lower emission.

### 2.4 Piezofluorochromic Behaviors of AIE MOFs

**Figure 5:**
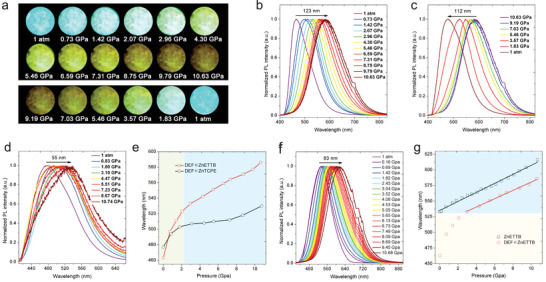
a) Photographs of DEF ∈ ZnETTB crystals under UV irradiation upon increasing or reducing hydrostatic pressure. b) Solid‐state fluorescence spectra of DEF ∈ ZnETTB upon increasing hydrostatic pressure from 1 atm (101 kPa) to 10.63 GPa. c) Solid‐state fluorescence spectra of DEF ∈ ZnETTB upon reducing hydrostatic pressure. d) Solid‐state fluorescence spectra of DEF ∈ ZnTCPE upon increasing hydrostatic pressure from 1 atm (101 kPa) to 10.74 GPa. e) Piezofluorochromic comparison of DEF ∈ ZnTCPE and DEF ∈ ZnETTB. f) Solid‐state fluorescence spectra of crystal ZnETTB upon increasing hydrostatic pressure from 1 atm (101 kPa) to 10.68 GPa. g) Piezofluorochromic comparison of DEF ∈ ZnETTB and ZnETTB.

DEF ∈ ZnETTB with elongated ligands also possesses higher elasticity over DEF ∈ ZnTCPE, which should show specific piezofluorochromic behavior to show emission change as a result of geometry distortion in response to external pressure. The piezofluorochromic behavior of DEF ∈ ZnETTB was investigated in the hydrostatic pressure experiment with a diamond anvil cell (DAC) device (**Figure** **5**a).^[^ ^35^, ^36^ ^]^ With the pressure increasing from 1 atm (101 kPa) to 10.63 GPa, the crystals changed from bright blue fluorescence to dim yellowish emission under 365 nm light (Figure 5a). Solid‐state emission spectra of DEF ∈ ZnETTB further confirmed the redshifted emission peaks from 463 to 586 nm along with gradually decreased brightness, upon increasing the hydrostatic pressure (Figure 5b; Figure S18a, Supporting Information). Intriguingly, the emission of DEF ∈ ZnETTB could be gradually restored after decompression (Figure 5a,c; Figure S18b, Supporting Information), but cannot be fully recovered to its original state under 1 atm conditions, which may be caused by the loss of some free solvent molecules. Nevertheless, it can still be explained that the DEF ∈ ZnETTB frame remains stable under the above‐mentioned high‐pressure conditions. In addition, the Raman spectrum of DEF ∈ ZnETTB shows no obvious change in the absorption peaks at 408, 785, 1000, 1132, and 1606 cm^−1^, as the pressure gradually increases, further indicating its frame stability during compression and decompression processes (Figure S19, Supporting Information). DEF ∈ ZnTCPE also showed certain piezofluorochromic behavior with high frame stability under high pressure (Figures S20 and S21, Supporting Information). However, it only showed a 53 nm redshift in emission peaks under 10.74 GPa (Figure 5d), much smaller than that of DEF ∈ ZnETTB (123 nm) (Figure 5e). This should be attributed to the higher structural elasticity of DEF ∈ ZnETTB due to its elongated ETTB^8−^ ligand. Therefore, increasing the length of AIEgens is an effective strategy for enhancing the elasticity of AIE MOFs to achieve more sensitive thermal‐ and pressure‐responsive performance.

We hypothesize that the higher piezofluorochromic sensitivity of DEF ∈ ZnETTB especially at low‐pressure ranges (1 atm to 3 GPa) is related to the presence of DEF solvent molecules. The piezofluorochromic behavior of ZnETTB was further evaluated. During the gradual increase of hydrostatic pressure from 1 atm to 10.68 GPa, maximum solid‐state emission of ZnETTB gradually redshifted from 533 to 616 nm (Figure 5f; Figure S22, Supporting Information). It is worth noting that the solid‐state fluorescence of ZnETTB can be completely restored upon restoring the pressure to 1 atm (Figures S22b and S23, Supporting Information), indicating the excellent elasticity and stability of ZnETTB. It also proves that the nonfully recovered emission for DEF ∈ ZnETTB is indeed caused by the loss of DEF solvent molecules. The longer emission wavelength of ZnETTB over DEF ∈ ZnETTB under different high pressures shall be attributed to its more distorted ETTB^8−^ ligand. High pressure leads to more distortion and, hence, more redshifted emission. DEF ∈ ZnETTB showed a better piezofluorochromic performance over ZnETTB, and their emission peak shifts in tested pressure range are 123 and 83 nm, respectively. Such improved performance was mainly attributed to higher sensitivity of DEF ∈ ZnETTB at low‐pressure range. The absolute sensitivity of DEF ∈ ZnETTB (29.98 nm GPa^−1^) at low pressure is much higher than that of 2D perovskite materials (14.4 nm GPa^−1^), showing great potential as an ultrasensitive pressure sensor.^[^ ^36^ ^]^ ZnETTB showed linear correlations between emission peaks and hydrostatic pressure at all tested ranges (Figure 5g). Such a linear correlation could also be observed for DEF ∈ ZnETTB with a similar slope when the pressure is above 3 GPa, indicating their similar piezofluorochromic behavior at high pressures, while DEF ∈ ZnETTB is much more sensitive to pressure change at low‐pressure range (below 3 GPa). A 71 nm redshift of emission peak was observed for DEF ∈ ZnETTB when increasing pressure from 1 atm to 3 GPa, while the value for ZnETTB is only 23 nm. Therefore, both the structural elasticity and the specific amide interaction are responsible for improved piezofluorochromic behavior of DEF ∈ ZnETTB. The piezofluorochromic behavior of DEF ∈ ZnETTB under low‐pressure conditions is mainly caused by the loss of solvent molecules in the pores, while the piezofluorochromic behavior under high‐pressure conditions is attributed to the elasticity of the ZnETTB frame.

## 3 Conclusions

In summary, we report that elongating AIE rotor ligands could render the newly formed AIE MOF (ZnETTB) with more elasticity, more space for intramolecular motion, and specific amide‐sensing capability. ZnETTB with 3D topology is constricted with elongated H_8_ETTB ligand, which provides sufficient void space for intramolecular motions of ETTB^8−^ ligand, and ZnETTB showed redshifted and weaken emission rather than enhanced ones as compared to its H_8_ETTB ligand. Such unique topology and molecular geometry afford ZnETTB the specific guest–host interaction with amide, where DEF, as an example, is anchored through C—H···O and C—H···*π* bonds. DEF anchoring reduces the distortion level and restricts the intramolecular motions of ETTB^8−^ ligand; as a consequence, ZnETTB showed blueshifted and intensified emission upon specific interaction of amide. Moreover, amide anchoring also affords the Solv. ∈ ZnETTB with the excellent thermofluorochromic behavior as well as the enhanced piezofluorochromic sensitivity on the basis of its elastic framework. We believe that this is one of the very rare examples of amide‐responsive smart materials, and amide‐assisted thermofluorochromic and piezofluorochromic behaviors. This work manifests the unique advantages of utilizing twisted AIE rotors for constructing of novel MOFs with new topology and responsiveness. It shall open up new windows to design more superior intelligent MOFs from the molecular structure levels.

## Conflict of Interest

The authors declare no conflict of interest.

## Supporting information

Supporting Information

## Data Availability

The data that support the findings of this study are available in the supplementary material of this article.
