# Supplementary material for: Smart Tetraphenylethene‐Based Luminescent Metal–Organic Frameworks with Amide‐Assisted Thermofluorochromics and Piezofluorochromics
Source: Adv Sci (Weinh). 2022 Mar 31;9(16):2200850. doi: 10.1002/advs.202200850 (PMC9165507; doi:10.1002/advs.202200850)
Supplement: Supplementary file 1 — Supporting Information [file ADVS-9-2200850-s001.pdf]

ADVANCED SCIENCE
Open Access

Supporting Information

for *Adv. Sci.*, DOI 10.1002/advs.202200850

Smart Tetraphenylethene-Based Luminescent Metal–Organic Frameworks with Amide-Assisted Thermofluorochromics and Piezofluorochromics

Zhong-Hong Zhu, Changjiang Bi, Hua-Hong Zou, Guangxue Feng, Shuping Xu* and Ben Zhong Tang**

Supporting Information

Smart Tetraphenylethene-Based Luminescent Metal-Organic Frameworks with Amide-Assisted Thermofluorochromics and Piezofluorochromics

Zhong-Hong Zhu,[a,†] Changjiang Bi,[b,†] Guangxue Feng,*[a] Hua-Hong Zou,[c] Shuping Xu,*[b] and Ben Zhong Tang*[a,d]

[a] AIE Institute, State Key Laboratory of Luminescent Materials and Devices, Guangdong Provincial Key Laboratory of Luminescence from Molecular Aggregates, School of Materials Science and Engineering, South China University of Technology, Guangzhou, 510640, China

[b] State Key Laboratory of Supramolecular Structure and Materials, Institute of Theoretical Chemistry, College of Chemistry, Jilin University, Changchun 130012, China

[c] State Key Laboratory for Chemistry and Molecular Engineering of Medicinal Resources, School of Chemistry and Pharmacy of Guangxi Normal University, Guilin 541004, China

[d] Shenzhen Institute of Aggregate Science and Engineering, School of Science and Engineering, The Chinese University of Hong Kong, Shenzhen 518172, China

Table of Contents:

Experimental Section	
**Supporting Tables**	
**Table S1**	Crystallographic data of the ZnTCPE.
**Table S2**	Selected bond lengths (Å) and angles (°) of ZnTCPE.
**Table S1**	Crystallographic data of the ZnETTB.
**Table S2**	Selected bond lengths (Å) and angles (°) of ZnETTB.
**Table S3**	Crystallographic data of the DEF∈ZnETTB.
**Table S4**	Selected bond lengths (Å) and angles (°) of DEF∈ZnETTB.
**Supporting Figures**	
**Figure S1**	The simulated and experimental powder X-ray diffraction (PXRD) spectra of Solv.∈ZnTCPE.
**Figure S2**	TG and DTG curves of Solv.∈ZnTCPE.
**Figure S3**	Solid-state fluorescence spectra of organic ligand H_4TCPE, Solv.∈ZnTCPE and ZnTCPE under 365 nm excitation, respectively.
**Figure S4**	The simulated and experimental PXRD spectra of Solv.∈ZnETTB.
**Figure S5**	Infrared (IR) spectrum of Solv.∈ZnETTB.
**Figure S6**	SEM images of Solv.∈ZnETTB.
**Figure S7**	TG and DTG curves of Solv.∈ZnETTB.
**Figure S8**	(a) N_2 adsorption isotherm of ZnETTB at 77 K. (b) Pore size distribution of ZnETTB obtained under N_2 adsorption conditions.
**Figure S9**	Solid-state fluorescence spectra of Solv.∈ZnETTB, ZnETTB, H_8ETTB and ZnETTB after stored at room temperature for 1 month, respectively.
**Figure S10**	Fluorescence lifetimes of Solv.∈ZnTCPE (a) and ZnTCPE (b).
**Figure S11**	Chromaticity coordinate (CIE) of thermochromic solid-state fluorescence of Solv.∈ZnETTB.
**Figure S12**	UV-Vis absorption spectra of Solv.∈ZnETTB and ZnETTB, respectively.
**Figure S13**	Infrared (IR) spectra of Solv.∈ZnETTB and ZnETTB.
**Figure S14**	PXRD spectra of Solv.∈ZnETTB upon treated by different temperatures.
**Figure S15**	Cyclic switching of the solid-state fluorescence emission wavlength and intensity of Solv.∈ZnETTB and ZnETTB.
**Figure S16**	PXRD of ZnETTB crystals after soaking in different organic solvents.
**Figure S17**	Fluorescence lifetime of DEF∈ZnETTB and ZnETTB.
**Figure S18**	(a) Solid-state fluorescence spectra of crystal DEF∈ZnETTB upon increasing hydrostatic pressure from 1 atm (101 kPa) to 10.63 GPa. (b) Solid-state fluorescence spectra of DEF∈ZnETTB upon reducing hydrostatic pressure.
**Figure S19**	Raman spectra of DEF∈ZnETTB crystals upon increasing hydrostatic pressure from 1 atm (101 kPa) to 10.72 GPa.
**Figure S20**	(a) Photographs of DEF∈ZnTCPE crystals under UV irradiation upon increasing or reducing hydrostatic pressure. (b) Solid-state fluorescence spectra of crystal

	DEF∈ZnTCPE upon increasing hydrostatic pressure from 1 atm (101 kPa) to 10.74 GPa. (c) Fluorescence spectra of DEF∈ZnTCPE upon reducing hydrostatic pressure.
**Figure S21**	Raman spectra of DEF∈ZnTCPE crystals upon increasing hydrostatic pressure from 1 atm (101 kPa) to 10.10 GPa.
**Figure S22**	Photographs of the ZnETTB crystals under UV irradiation upon (a) increasing or (b) reducing hydrostatic pressure.
**Figure S23**	Solid-state fluorescence spectra of ZnETTB upon reducing hydrostatic pressure.

Experimental Section

Materials and Measurements.

All reagents were obtained from commercial sources and used without further purification. Both 4,4',4'',4'''-(ethene-1,1,2,2-tetrayl)tetrabenzoic acid (H_4TCPE) and 4',4''',4''''',4'''''''-(ethene-1,1,2,2-tetrayl)tetrakis(([1,1'-biphenyl]-3,5-dicarboxylic acid)) (H_8ETTB) were purchased from Bide Pharmatech Ltd (Shanghai, China). All organic solvents were purchased at Macklin (Shanghai, China). Elemental analyses for C, and H were performed on a vario MICRO cube. Infrared spectra were recorded by transmission through KBr pellets containing *ca.* 0.5% of the MOFs using a PE Spectrum FT-IR spectrometer (400-4,000 cm^{-1}). Thermogravimetric analyses (TGA) were conducted in a flow of nitrogen at a heating rate of 5 °C/min using a NETZSCH TG 209 F3. Powder X-ray diffraction (PXRD) spectra were recorded on either a D8 Advance (Bruker) diffractometer at 293 K (Cu-Kα). The samples were prepared by crushing crystals and the powder placed on a grooved aluminum plate. Diffraction patterns were recorded from 2º to 55º at a rate of 5º min^{-1}. Ultraviolet-visible absorption spectra were recorded on a Shimadzu UV-2600 spectrophotometer, and fluorescence spectra were recorded on a Horiba Fluoromax-4 and FLS980 fluorescence spectrophotometer.

Single-crystal X-ray crystallography.

Diffraction data for the complex were collected on a Bruker SMART CCD diffractometer (Cu-Kα radiation and λ = 1.54184 Å) in Φ and ω scan modes. The structures were solved by direct methods, followed by difference Fourier syntheses, and then refined by full-matrix least-squares techniques on F^2 using *SHELXL and Olex2*.[1] All other non-hydrogen atoms were refined with anisotropic thermal parameters. Hydrogen atoms were placed at calculated positions and isotropically refined using a riding model. Tables S1 ~ S6 summarizes X-ray crystallographic data and refinement details for the all MOFs. The CCDC reference numbers are 2143081 (DEF∈ZnETTB), 2143083 (ZnTCPE), and 2143116 (ZnETTB).

Synthesis of Solv.∈ZnTCPE and Solv.∈ZnETTB.

Solv.∈ZnTCPE: $Zn(NO_3)_2{\cdot}6H_2O$ (29.8 mg, 0.1 mmol) and H_4TCPE (10 mg, 0.02 mmol) in a mixed solvent of DEF (1 mL) and EtOH (1 mL) were placed in a sealed vial (20 mL) and heated up to and maintained at 80 °C for 48 h. Colorless needle crystals were collected, after being washed by

DEF. The yield is about 51.3% (calculated with the amount of ligand H_4TCPE).[2]

Solv.∈ZnETTB: $Zn(NO_3)_2 \cdot 6H_2O$ (29.8 mg, 0.1 mmol) and H_8ETTB (24 mg, 0.02 mmol) in a mixed solvent of DEF (1 mL) and H_2O (1 mL) were placed in a sealed vial (20 mL) and heated up to and maintained at 85 °C for 48 h. Colorless needle crystals were collected, after being washed by DEF. The yield is about 81.6% (calculated with the amount of ligand H_8ETTB). Elemental analysis theoretical value [$C_{29}H_{18}O_{10}Zn_2 \cdot (H_2O)_3(C_5H_{11}NO)_2$]: C, 51.28%; H, 5.08%; N, 3.07%; experimental value: C, 51.27%; H, 5.02%; N, 3.03%.

ZnTCPE: Put Solv.∈ZnTCPE in a vacuum drying oven set to 160 °C and keep it for 2 h.

ZnETTB: Put Solv.∈ZnETTB in a vacuum drying oven set to 160 °C and keep it for 2 h.

DEF∈ZnETTB: Soak the activated ZnETTB in DEF for 2 h.

***In Situ* High-Pressure Optical Experiments.**

All of the *in situ* high-pressure experiments shown in this study were carried out with a symmetric DAC apparatus. II-type ultralow fluorescence diamonds with a culet size of 300 μm were used. A T301 stainless steel gasket was pre-indented by the diamonds and was drilled to generate a 100 μm diameter cavity for loading the samples. Typically, the prepared DEF∈ZnETTB, DEF∈ZnTCPE and ZnETTB crystals were enclosed in the gasket hole together with a ruby ball for determining the actual pressure through the standard ruby fluorescent technique. Therein, silicon oil with a viscosity of 10 cSt was utilized as the pressure transmitting medium (PTM), which was purchased from Dow Corning Corporation (South Saginaw Road, Midland, MI, USA). The high-pressure evolution of steady-state PL spectra was collected utilizing a modified spectrophotometer (Ocean Optics, QE65000). All the high-pressure experiments were conducted at room temperature.

Reference

1. Sheldrick, G. M. *Acta Crystallogr., Sect. C: Struct. Chem.* **2015**, *71*, 3-8.
2. Shustova, N. B.; McCarthy, B. D.; Dincă, M. Turn-On Fluorescence in Tetraphenylethylene-Based Metal–Organic Frameworks: An Alternative to Aggregation-Induced Emission. *J. Am. Chem. Soc.* **2011**, *133* (50), 20126–20129.

Table S1. Crystallographic data of the ZnTCPE.

	ZnTCPE
Formula	$Zn_2C_{50}H_{64}N_4O_{14}$
Formula weight	1075.79
T, K	293 K
Crystal system	Monoclinic
Space group	$P2_1/c$
a, Å	21.7663 (10)
b, Å	13.6252 (6)
c, Å	17.6526 (12)
α, º	90
β, º	90
γ, º	90
V, Å^3	5235.2 (5)
Z	23
D_c, g cm^{-3}	1.733
μ, mm^{-1}	2.66
F(000)	2783
2θ range for data collection/°	5.62 to 50
Reflns coll.	18925, 8595
Unique reflns R_{int}	
Observed data [$I > 2\sigma(I)$]	4648
N_{par}, N_{ref}	631,8595
R_1^a ($I > 2\sigma(I)$)	0.1029

$^aR_1 = \Sigma||F_o|-|F_c||/\Sigma|F_o|$, $^bwR_2 = [\Sigma w(F_o^2-F_c^2)^2/\Sigma w(F_o^2)^2]^{1/2}$

Table 2. Selected bond lengths (Å) and angles (°) of ZnTCPE.

**Bond lengths (Å)**					
Zn01—O004	1.858(5)	Zn01—O00C^{iii}	1.906(5)	Zn02—O006^{ii}	1.869(5)
Zn01—O007^{i}	1.868(5)	Zn02—O003^{iv}	1.900(5)	Zn02—O008	2.452(7)
Zn01—O00A	2.465(7)	Zn02—O005^{v}	1.871(5)	Zn02—O009	1.916(5)
Zn01—O00B^{ii}	1.894(5)				
**Bond angles (°)**					
O004—Zn01—O007^{i}	162.4(3)	O00B^{ii}—Zn01—O00A	108.1(2)	O005^{v}—Zn02—O009	75.5(2)

O004—Zn01—O00A	97.9(2)	O00B^{ii}—Zn01—O00C^{iii}	142.2(3)	O006^{ii}—Zn02—O003^{iv}	73.5(2)
O004—Zn01—O00B^{ii}	98.5(3)	O00C^{iii}—Zn01—O00A	109.7(3)	O006^{ii}—Zn02—O005	161.5(3)
O004—Zn01—O00C^{iii}	75.0(2)	O003^{iv}—Zn02—O008	108.5(2)	O006^{ii}—Zn02—O008	99.9(3)
O007^{i}—Zn01—O00A	99.5(3)	O003^{iv}—Zn02—O009	141.4(3)	O006^{ii}—Zn02—O009	100.8(3)
O007^{i}—Zn01—O00B^{ii}	73.7(2)	O005^{v}—Zn02—O003^{iv}	97.9(3)	O009—Zn02—O008	110.0(3)
O007^{i}—Zn01—O00C^{iii}	101.3(3)	O005^{v}—Zn02—O008	98.4(3)		

Table S3. Crystallographic data of the ZnETTB.

	ZnETTB
Formula	$C_{58}H_{28}O_{20}Zn_4$
Formula weight	1298.32
T, K	105 K
Crystal system	Tetragonal
Space group	P-42c
a, Å	19.2489(2)
b, Å	19.2489(2)
c, Å	34.9632(5)
α, °	90
β, °	90
γ, °	90
V, Å^3	12954.6(3)
Z	4
D_c, g cm^{-3}	0.666
μ, mm^{-1}	1.11
F(000)	2608
2θ range for data collection/°	5.24 to 134.152
Reflns coll.	30199, 10426
Unique reflns R_{int}	
Observed data [$I > 2\sigma(I)$]	6622
N_{par}, N_{ref}	372,10426
R_1^a ($I > 2\sigma(I)$)	0.0523

$^aR_1 = \Sigma||F_o|-|F_c||/\Sigma|F_o|$, $^bwR_2 = [\Sigma w(F_o^2-F_c^2)^2/\Sigma w(F_o^2)^2]^{1/2}$

Table S4. Selected bond lengths (Å) and angles (°) of ZnETTB.

**Bond lengths (Å)**

Zn01—O003^{i}	2.030(4)	Zn01—O00C	1.975(6)	Zn02—O00B^{iii}	2.009(4)
Zn01—O005	1.995(4)	Zn01—O00P	1.965(4)	Zn02—O00M^{iv}	2.063(4)
Zn01—O006^{ii}	2.012(5)	Zn02—O004	2.033(4)	Zn02—O007^{iii}	2.021(4)
Zn02—O008	1.980(4)				
**Bond angles (°)**					
O005—Zn01—O003^{i}	86.3(2)	O00P—Zn01—O00[illegible]	90.93(17)	O007^{iii}—Zn02—O0[illegible]	87.55(18)
O005—Zn01—O006^{ii}	87.01(19)	O00P—Zn01—O00[illegible]	160.5(16)	O008—Zn02—O00[illegible]	98.64(17)
O006^{ii}—Zn01—O003^{i}	156.06(16)	O00P—Zn01—O00[illegible]	87.8(2)	O008—Zn02—O00[illegible]	102.02(18)
O00C—Zn01—O003^{i}	98.3(2)	O00P—Zn01—O00[illegible]	103.8(2)	O00B^{iii}—Zn02—O0[illegible]	88.09(18)
O00C—Zn01—O005	95.7(2)	O004—Zn02—O00[illegible]	155.44(15)	O00B^{iii}—Zn02—O0[illegible]	159.53(15)
O00C—Zn01—O006^{ii}	105.2(2)	O007^{iii}—Zn02—O0[illegible]	90.5(2)	O00B^{iii}—Zn02—O0[illegible]	85.3(2)
O008—Zn02—O007^{iii}	101.60(17)	O008—Zn02—O00[illegible]	102.34(18)		

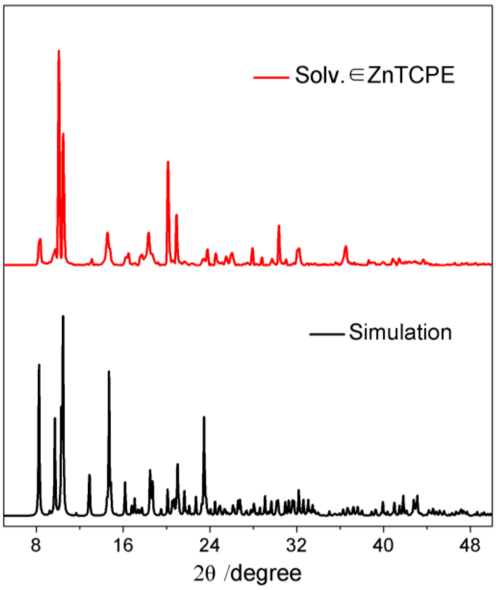

Figure S1. The simulated and experimental powder X-ray diffraction (PXRD) spectra of Solv.∈ZnTCPE.

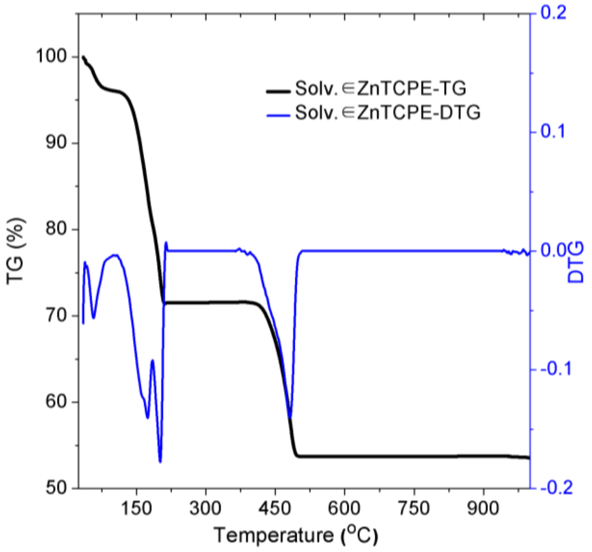

Figure S2. TG and DTG curves of Solv.∈ZnTCPE.

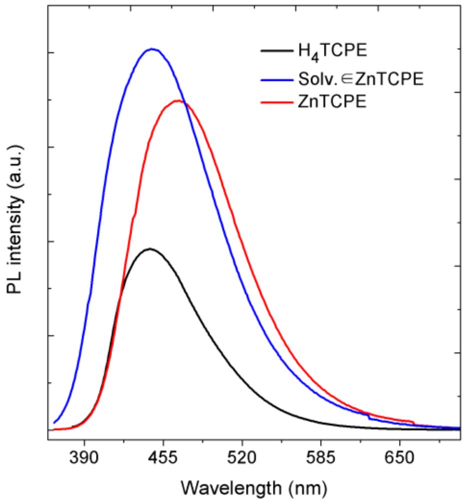

Figure S3. Solid-state fluorescence spectra of organic ligand H_4TCPE, Solv.∈ZnTCPE and ZnTCPE under 365 nm excitation, respectively.

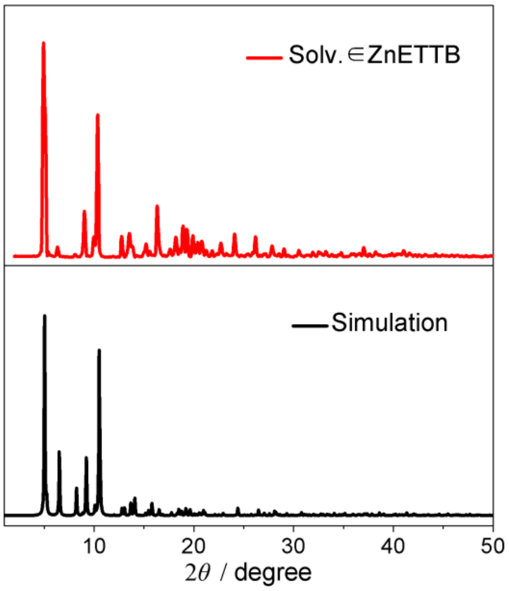

Figure S4. The simulated and experimental PXRD spectra of Solv.∈ZnETTB.

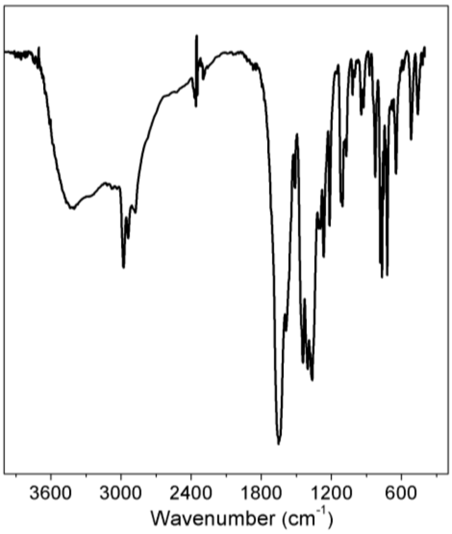

Figure S5. Infrared (IR) spectrum of Solv.∈ZnETTB.

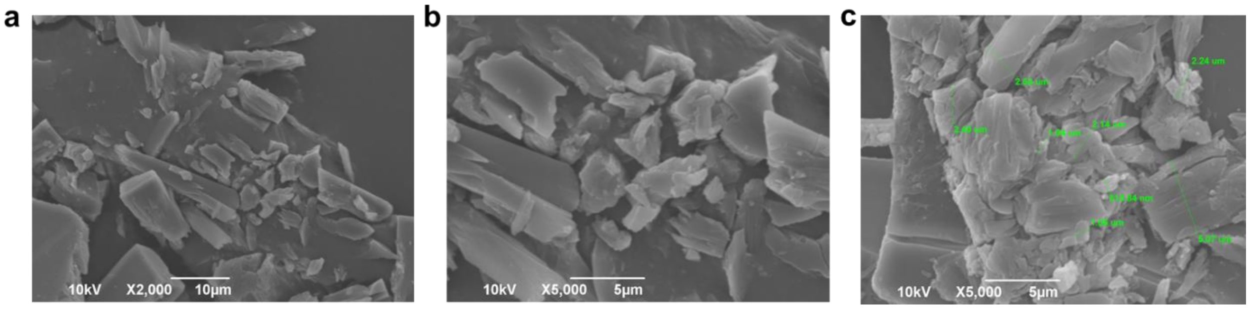

Figure S6. SEM images of Solv.∈ZnETTB.

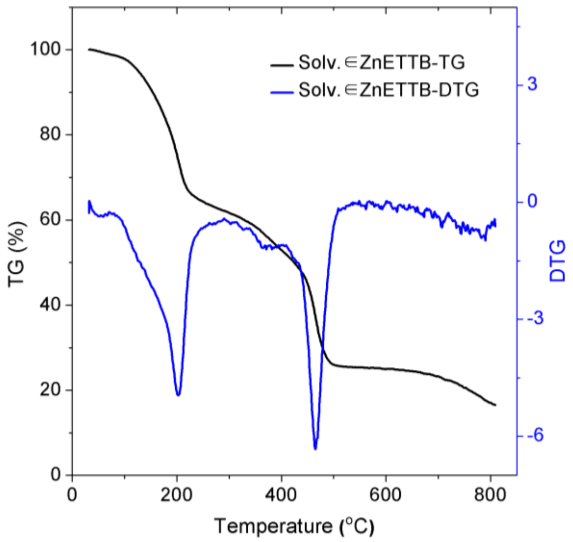

Figure S7. TG and DTG curves of Solv.∈ZnETTB.

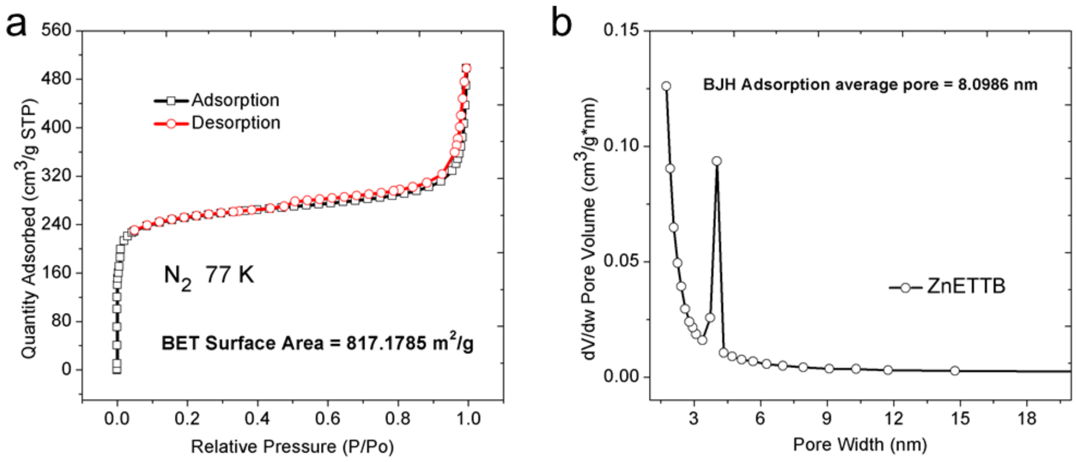

Figure S8. (a) N_2 adsorption isotherm of ZnETTB at 77 K. (b) Pore size distribution of ZnETTB

obtained under N_2 adsorption conditions.

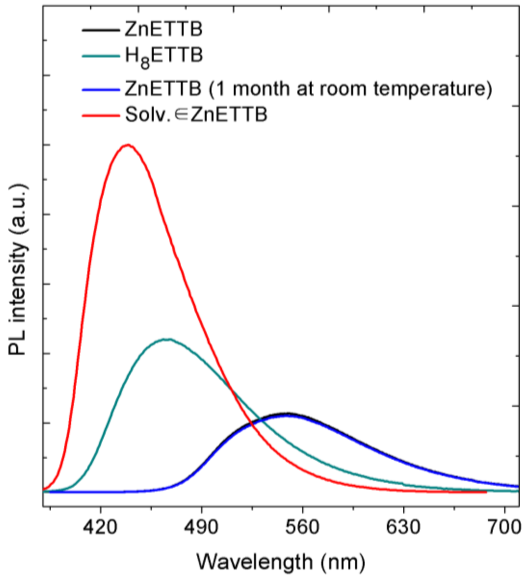

Figure S9. Solid-state fluorescence spectra of Solv.∈ZnETTB, ZnETTB, H_8ETTB and ZnETTB after stored at room temperature for 1 month, respectively.

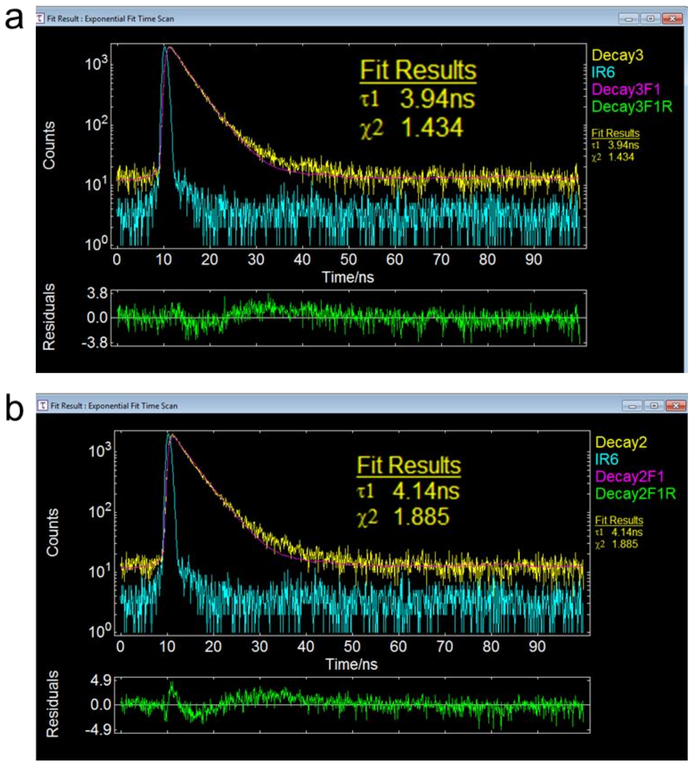

Figure S10. Fluorescence lifetimes of Solv.∈ZnTCPE (a) and ZnTCPE (b).

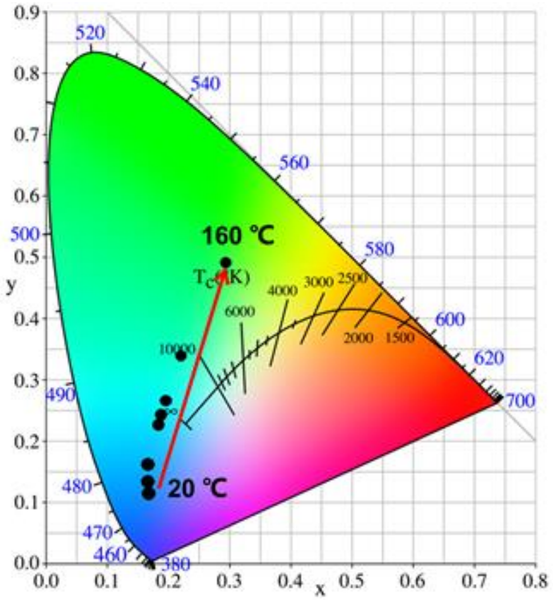

Figure S11. Chromaticity coordinate (CIE) of thermochromic solid-state fluorescence of Solv.∈ZnETTB.

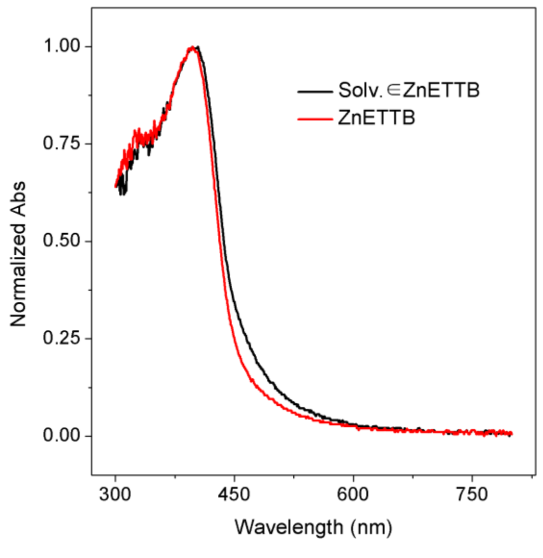

Figure S12. UV-Vis absorption spectra of Solv.∈ZnETTB and ZnETTB, respectively.

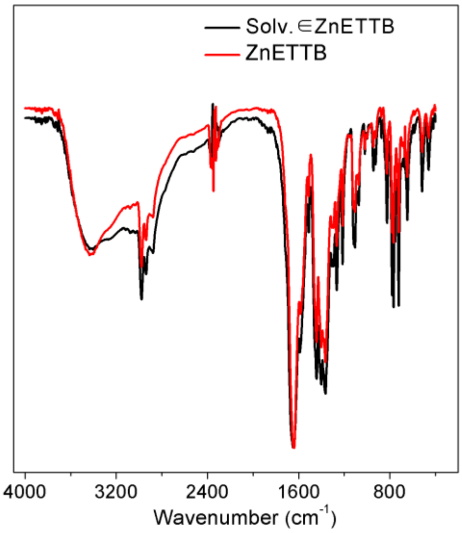

Figure S13. Infrared (IR) spectra of Solv.∈ZnETTB and ZnETTB.

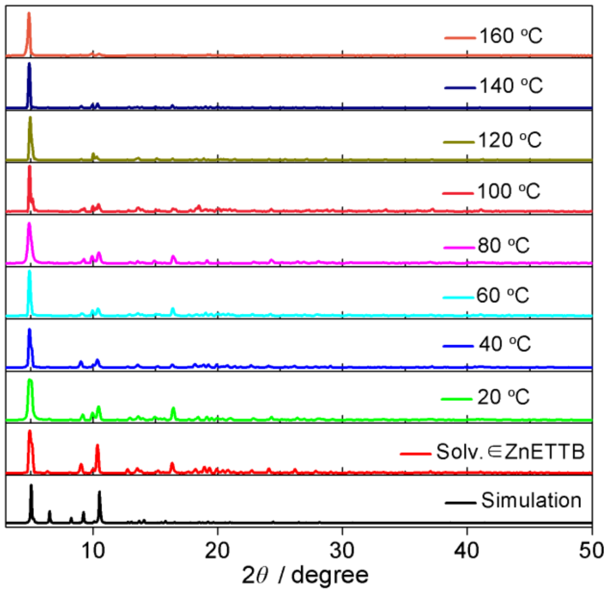

Figure S14. PXRD spectra of Solv.∈ZnETTB upon treated by different temperatures.

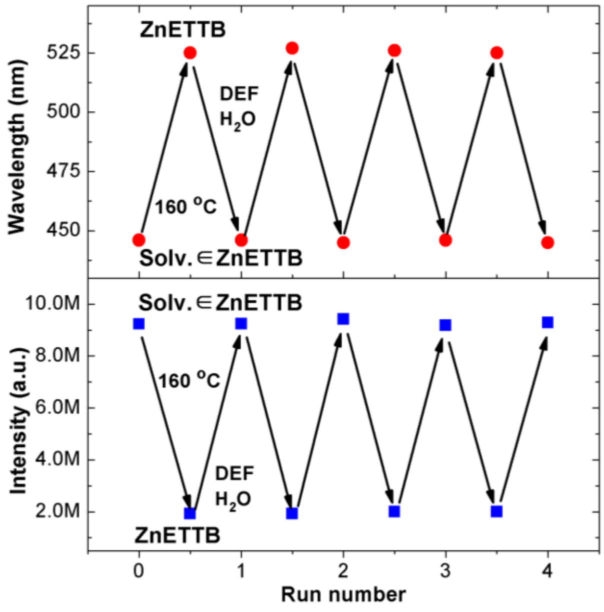

Figure S15. Cyclic switching of the solid-state fluorescence emission wavlength and intensity of Solv.∈ZnETTB and ZnETTB.

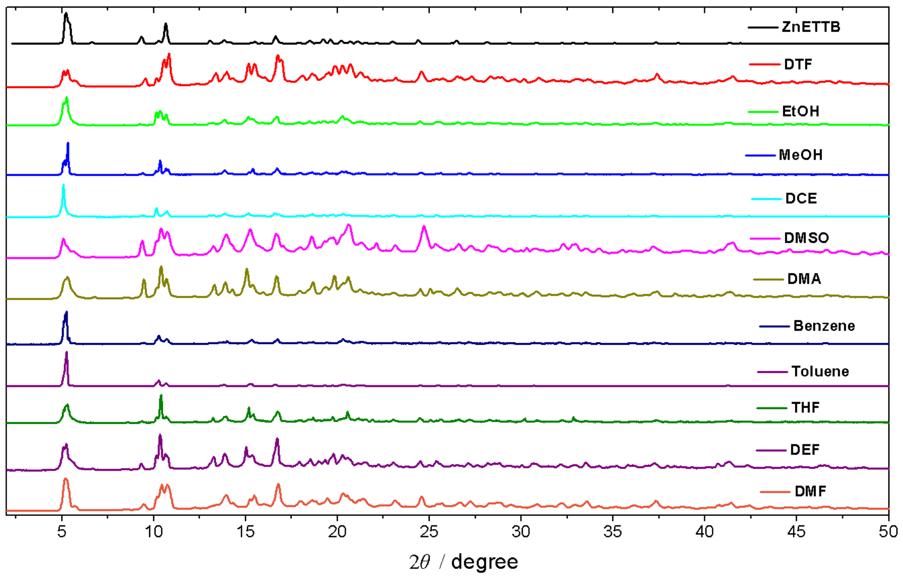

Figure S16. PXRD of ZnETTB crystals after soaking in different organic solvents.

Table S5. Crystallographic data of the DEF∈ZnETTB.

	DEF∈ZnETTB
Formula	$C_{39}H_{27}O_{12}N_2Zn_2$
Formula weight	857.45
T (K)	100 K
Crystal system	Tetragonal
Space group	$P4_2/mnm$
a (Å)	19.2251(2)
b (Å)	19.2251(2)
c (Å)	35.0562(4)
α (°)	90
β (°)	90
γ (°)	90
V (Å^3)	12956.9(3)
Z	8
D_c(g cm^{-3})	0.879
μ (mm^{-1})	1.24
Reflns coll.	27501
Unique reflns	6084
R_{int}	0.020
$^aR_1[I \geq 2\sigma(I)]$	0.0931
$^bwR_2$(all data)	0.3391
GOF	1.094

$^aR_1 = \Sigma||F_o|-|F_c||/\Sigma|F_o|$, $^bwR_2 = [\Sigma w(F_o^2-F_c^2)^2/\Sigma w(F_o^2)^2]^{1/2}$

Table S6. Selected bond lengths (Å) and angles (°) of DEF∈ZnETTB.

Bond lengths (Å)

Zn01—Zn02^{i}	3.0206(12)	Zn01—O005^{i}	2.032(4)	Zn01—O006	2.029(4)
Zn01—O005^{ii}	2.032(4)	Zn01—O006^{iii}	2.029(4)	Zn01—O007	1.960(5)
Zn02—O003^{iv}	2.038(4)	Zn02—O003^{v}	2.038(4)	Zn02—O004	2.025(4)
Zn02—O004^{vi}	2.025(4)	Zn02—O00E	1.991(6)		

Bond angles (°)

O005^{ii}—Zn01—O005^{i}	85.7 (3)	O007—Zn01—O006	102.4(2)	O006^{iii}—Zn01—O005^{i}	88.48(19)
O006—Zn01—O005^{ii}	157.72(17	O006^{iii}—Zn01—O005^{i}	157.72(17	O006—Zn01—O005^{i}	88.48(19)
O006^{iii}—Zn01—O006	88.82(3)	O007—Zn01—O005^{i}	99.7(2)	O007—Zn01—O006^{iii}	102.41(2)
O007—Zn01—O005^{ii}	99.71(2)	O003^{iv}—Zn02—O003^{v}	86.7(2)	O004—Zn02—O003^{iv}	157.7(17)
O004^{vi}—Zn02—O003^{iv}	87.92(18)	O004^{vi}—Zn02—O003^{v}	157.2(17)	O004—Zn02—O003^{v}	87.95(18)
O004^{vi}—Zn02—O004	88.82(3)	O00E—Zn02—O003^{v}	100.4(19)	O00E—Zn02—O004^{vi}	101.5(19)
O00E—Zn02—O004	101.52(19				

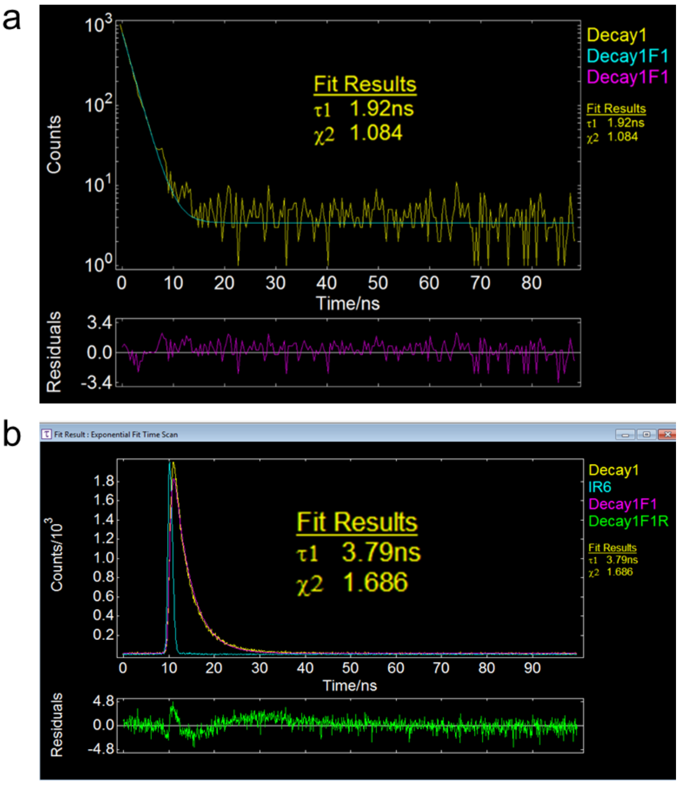

Figure S17. Fluorescence lifetime of DEF∈ZnETTB and ZnETTB.

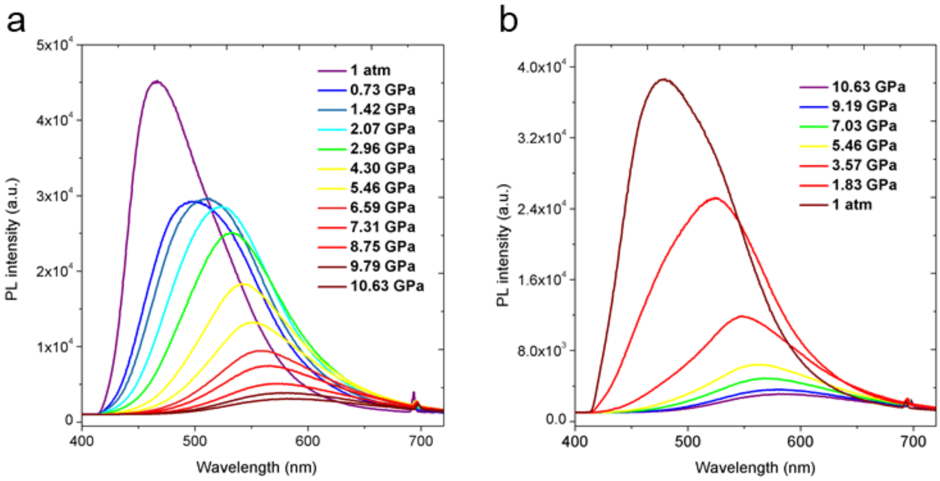

Figure S18. (a) Solid-state fluorescence spectra of crystal DEF∈ZnETTB upon increasing hydrostatic pressure from 1 atm (101 kPa) to 10.63 GPa. (b) Solid-state fluorescence spectra of DEF ∈ZnETTB upon reducing hydrostatic pressure.

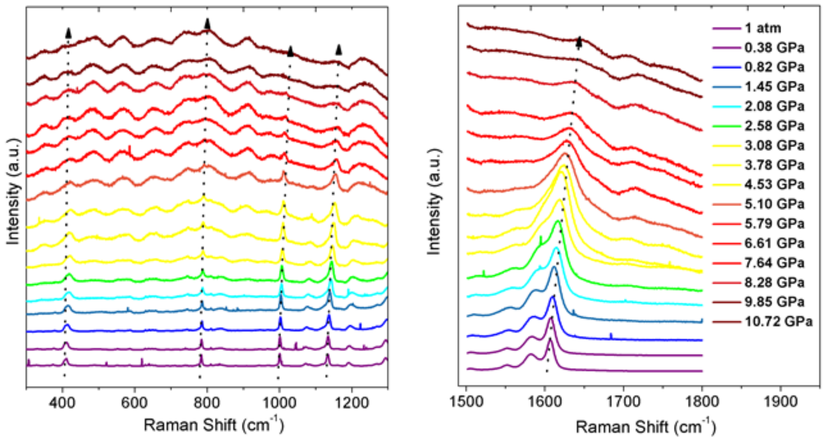

Figure S19. Raman spectra of DEF∈ZnETTB crystals upon increasing hydrostatic pressure from 1 atm (101 kPa) to 10.72 GPa.

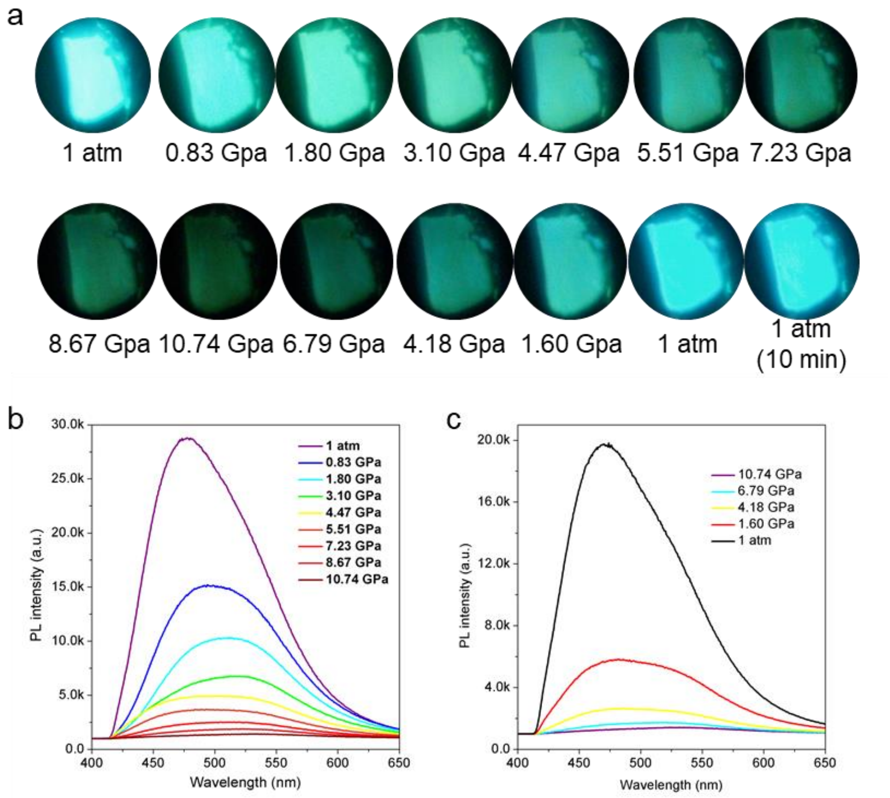

Figure S20. (a) Photographs of DEF∈ZnTCPE crystals under UV irradiation upon increasing or reducing hydrostatic pressure. (b) Solid-state fluorescence spectra of crystal DEF∈ZnTCPE upon increasing hydrostatic pressure from 1 atm (101 kPa) to 10.74 GPa. (c) Fluorescence spectra of DEF ∈ZnTCPE upon reducing hydrostatic pressure.

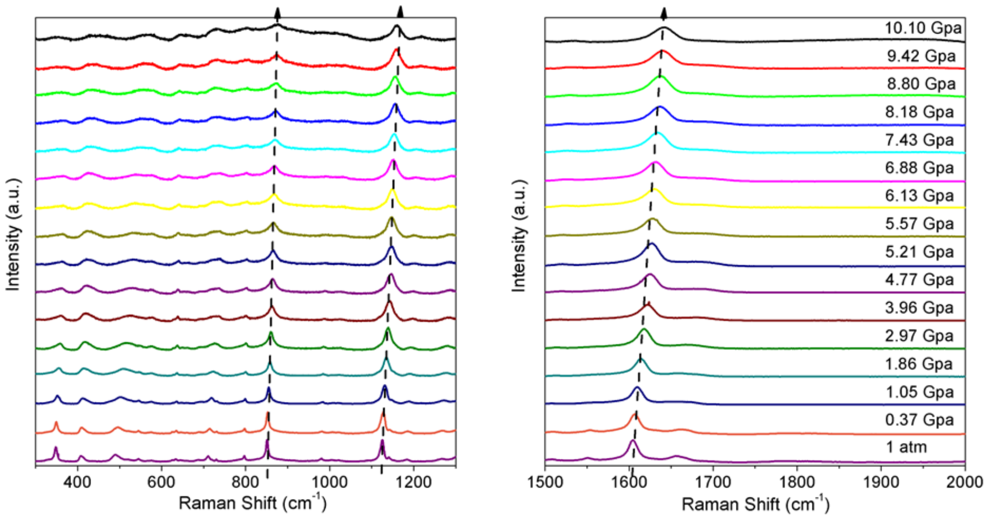

Figure S21. Raman spectra of DEF∈ZnTCPE crystals upon increasing hydrostatic pressure from 1

atm (101 kPa) to 10.10 GPa.

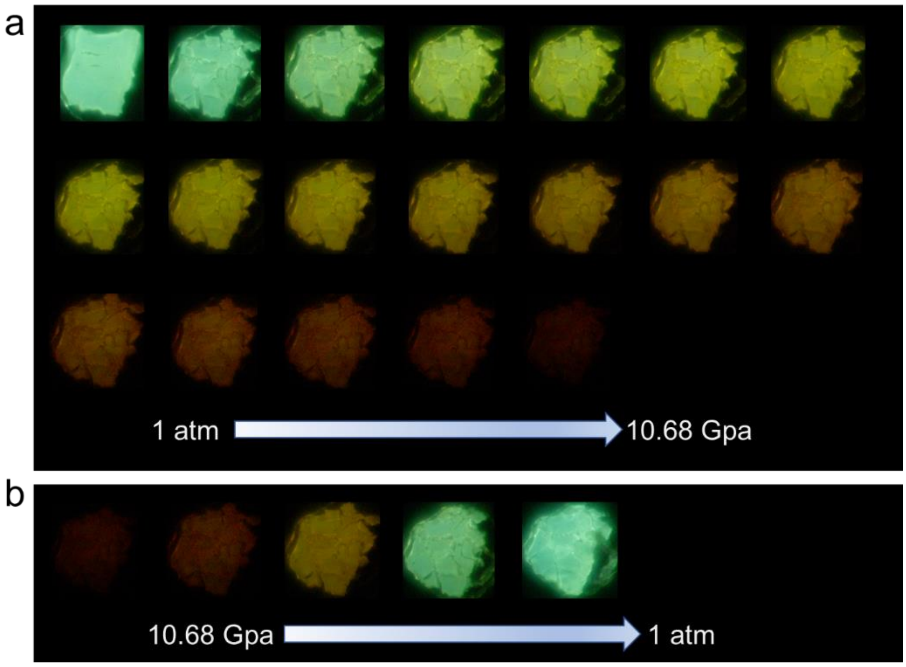

Figure S22. Photographs of the ZnETTB crystals under UV irradiation upon (a) increasing or (b) reducing hydrostatic pressure.

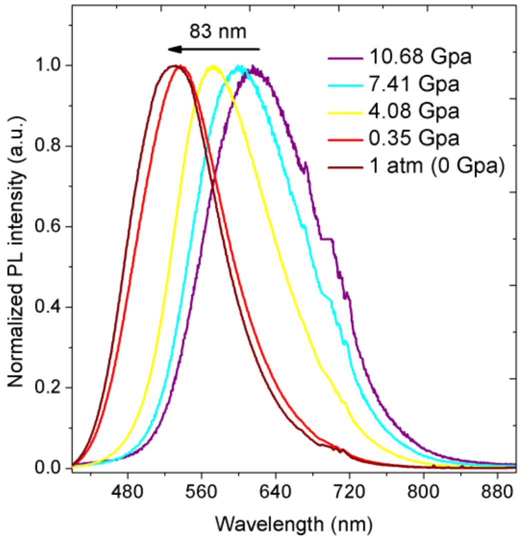

Figure S23. Solid-state fluorescence spectra of ZnETTB upon reducing hydrostatic pressure.
